# Supplementary material for: Inflammatory factors and risk of lung adenocarcinoma: a Mendelian randomization study mediated by blood metabolites
Source: Front Endocrinol (Lausanne). 2024 Aug 27;15:1446863. doi: 10.3389/fendo.2024.1446863 (PMC11384989; doi:10.3389/fendo.2024.1446863)
Supplement: Supplementary file 1 [file DataSheet1.zip › supplementary files/S3/GCST90274801_GCST90199955/forest.pdf]

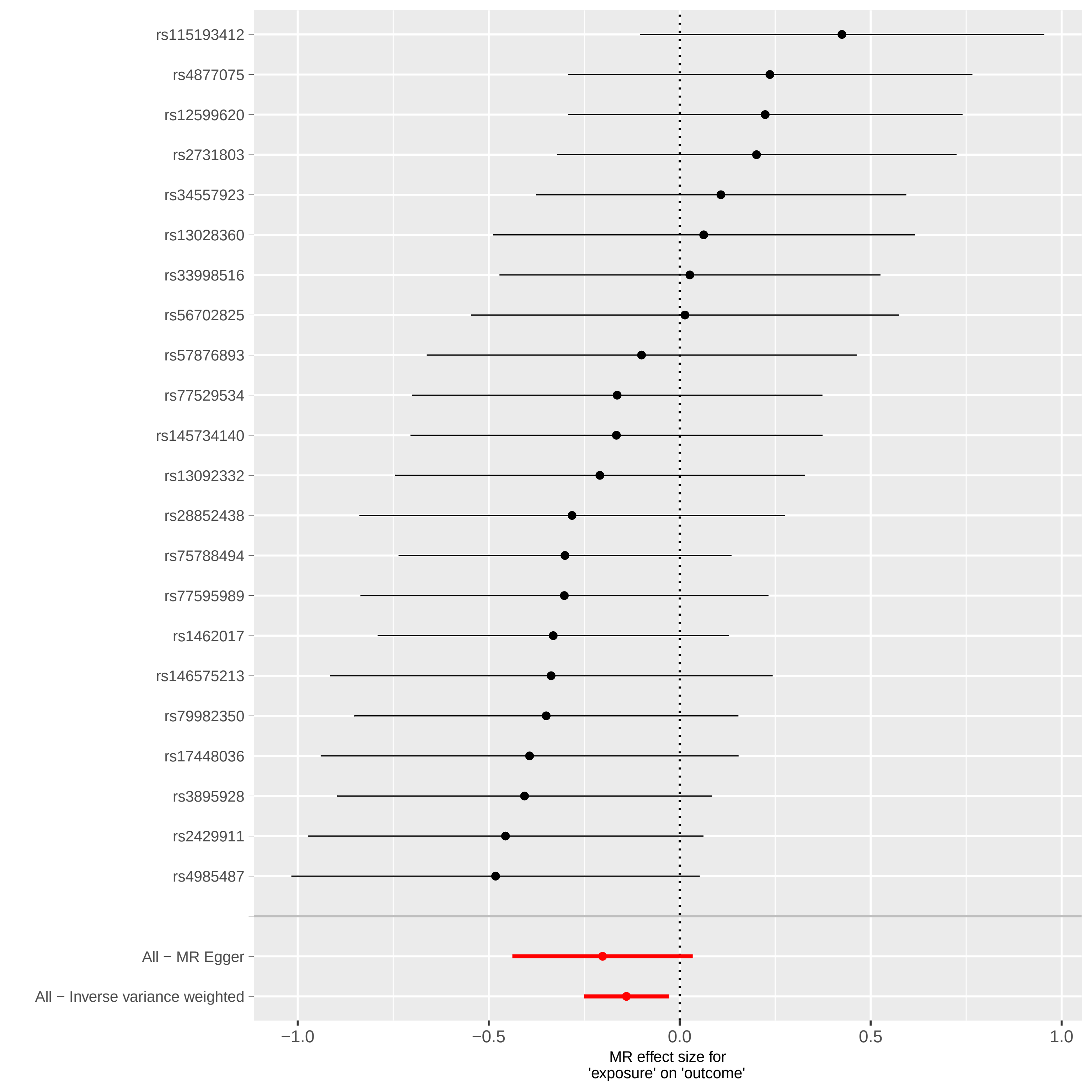

rs115193412
rs4877075
rs12599620
rs2731803
rs34557923
rs13028360
rs33998516
rs56702825
rs57876893
rs77529534
rs145734140
rs13092332
rs28852438
rs75788494
rs77595989
rs1462017
rs146575213
rs79982350
rs17448036
rs3895928
rs2429911
rs4985487
All − MR Egger
All − Inverse variance weighted
−1.0
−0.5
0.0
0.5
1.0
MR effect size for
'exposure' on 'outcome'
